# The case series of peritonitis due to perforated peptic ulcer: How does conservative management play role?

**DOI:** 10.1016/j.ijscr.2019.03.054

**Published:** 2019-04-05

**Authors:** Pongsatorn Asanasak

**Affiliations:** Department of Surgery, Songkhla Hospital, Muang, Songkhla, 90100, Thailand

**Keywords:** Conservative management, Non-operative treatment, Pneumoperitoneum-peritonitis, Perforated peptic ulcers

## Abstract

**Introduction:**

Surgical intervention is a conventional treatment for perforated peptic ulcer patients. This study aims to determine whether and how conservative non-operative management plays role in patients with pneumoperitoneum-peritonitis due to perforated peptic ulcers.

**Methods:**

A 9-year retrospective study was conducted in patients, who visit one surgeon service, with peritonitis due to perforated peptic ulcer and received non-operation conservative treatment. The treatment consists of nasogastric suction, intravenous fluid (IV) resuscitation, IV antibiotic and IV omeprazole. Outcomes and clinical course of conservative treatment in the selected group were reviewed. Factors associated with those outcomes and clinical course were analyzed.

**Results:**

There were 38 patients in this case series. Of which, 36 patients (94.7%) showed improvement after 24 h of conservative treatment and discharged without operation. Two patients underwent laparotomy in the 3^rd^ day of admission due to severe abdominal pain and progression of abdominal sign. There was no mortality in this case series. The conservative series had shorter hospital stay and lesser complication but prolong fever. Three factors indicated good outcomes in this series were found i.e. 1) free air in abdominal x-ray was not broader than the 1^st^ lumbar vertebral column height, 2) no free fluid seen in intra-peritoneal cavity by bedside ultrasound, 3) resuscitate fluid in the first 24 h was not more than 5 ml/kg/h.

**Conclusion:**

In this case series, conservative non-surgical management showed good results for patients with peptic ulcers perforation. It could be used as an effective alternative modality when carefully patient selection and closely observed.

## Highlights

• Minor hollow viscus perforation can recover by conservative management.
• It is challenge how to select patients.
• This study intends to propose criteria for case selection.
• The selective conservative management in patients with peritonitis and pneumoperitonium can achieve high successful rate.

## 1 Introduction

The accepted therapeutic options in pneumoperitoneum-peritonitis patients who were suspected perforated peptic ulcer was an operation. However, conservative management was essential in some situation [1,2] such as patient denied of operation, contraindication for operation or shortage of surgeon and facility. Conservative non-surgical management had been used in some places with various criteria and outcomes. Lay et al. [3] showed the mortality rate of conservative treatment was 40%, especially if patients had ≥Class IV of American Society of Anesthesiologists classification. Other group from France [4] found more than 50% of patients with perforated peptic ulcer responded to conservative treatment and demonstrated significant predictive factors associated with failure of the treatment as size of pneumoperitoneum, heart rate, pain at digital rectal exam and age >59 years. Patients’ conditions affected outcomes of the conservative management.

It is a challenge to select patients for better outcomes. We assume that minute ulcer might have higher rate of successful without operation. We, therefore, explored the role of conservative management in our case series of suspected perforated peptic ulcer in order to detect features that affect outcomes and clinical course.

## 2 Methods

A retrospective study from April 2009 to April 2017 was performed, at Surgical Department of our institute. Patients who, visited one surgeon service, presented with peritonitis due to suspected perforated peptic ulcer were enrolled in the study when they met the inclusion criteria. Conservative treatment was given to those patients. The proposed inclusion criteria for case selection were as follows: 1) free air in abdominal x-ray was not broader than the 1^st^ lumbar vertebral column height [4], 2) no free fluid seen in intra-peritoneal cavity by bedside ultrasound, 3) resuscitate fluid in the first 24 h not more than 5 ml/kg/h and 4) hemodynamic stable.

Conservative treatment consisted of intravenous fluid resuscitation, nasogastric tube with intermittent suction, intravenous administration of antibiotic (ceftriaxone 2 gm every 24 h metronidazole 500 mg every 8 h) and omeprazole 40 mg intravenous every 12 h. Vital sign, abdominal sign, urine output and effectiveness of stomach decompress were monitored. Outcome and clinical course of those studied patients were reviewed and analyzed. This work had been reported in line with the PROCESS criteria [5].

## 3 Results

Thirty eight patients met criteria and received conservative management in the case series study period. The clinical details were shown in Table 1 revealed male predominate (94.7%) with average age of 42 years (range 26–77). One-third of the studied patients had a history of previous peptic ulcer and 8 out of 38 patients used NSAIDS within 1 month before admitted to the hospital.

**Table 1:** Clinical characteristic of pneumoperitoneum-peritonitis patients who were suspected perforated peptic ulcers.

Characteristic	Number of patients
Male	36
Female	2
Mean age in years (range)	42 (26–77)
History of previous peptic ulcer	12
NSAIDS used (within 1 month before visit)^a^	8
Anti HIV + ve^b^	3
Hyperthyroidism	1

a NSAIDS = non-steroidal anti-inflammatory drugs.

b Anti HIV + ve = seropositive for human immunodeficiency virus.

In this series, 36 (94.7%) patients were successfully conservative managed. Only two patients failed and underwent laparotomy in the 3^rd^ day of admission due to severe pain and progress of abdominal signs. Both cases had chronic peptic ulcer perforation. Table 2 showed comparison between the successful- and failed-conservative groups in term of length of hospital stay and complications. It was found that the successful-conservative group stayed in hospital shorter than the failed-group at the average of 10 days compare to 14 days. However, the former group presented with pleural effusion and prolongs fever more than 7 days, while the latter group had none.

**Table 2:** The comparison of clinical characteristics, complications and outcomes of 38 patients with pneumoperitoneum-peritonitis due to perforated peptic ulcer.

Clinical characeristics/outcomes	Successful-conservative group (n = 36)	Failed-conservative group (n = 2)
Mean age in years (range)	47 (26–77)	48 (40–56)
Length of hospital stay in days (range)	10 (5–18)	14 (10, 18)
Surgical site infection	NA	0
Wound dehiscence/evisceration	NA	1
Pleural effusion	4	0
Prolong fever than 7 days	6	0
Intra-abdominal collection	0	0

NA = non-applicable.

Monitored parameters of the successful-conservative group were shown on Table 3. A group of successful conservative management (36 patients) showed improvement of symptoms and clinical signs in the 2^nd^ day after admission. All of the cases were discharged on good conditions which were no fever, no abdominal pain and well appetite. However, one patient with anti HIV +ve still had high grade fever but no abdominal pain and good appetite on the discharge date. His bedside ultrasound suggested no intra-abdominal collection. Home medications were continuing oral antibiotic for a week and oral omeprazole for 6 week course. Subsequent follow up, no complication was found in all patients. Pleural effusion in 4 patients was also disappeared in chest films on the 6 weeks follow up.

**Table 3:** Monitored parameters of successful conservative group.

Parameters	Average	Range
Volume of fluid resuscitation in the first 24 h (ml/kg/h)	2.4	2.2–3.8
Duration of fever (days)	4	2–10
Duration of NG tube decompression (days)	3.8	3–4

NG tube = Nasogastric Tube.

## 4 Discussion

In some situation conservative treatment of pneumoperitoneum-peritonitis due to peptic ulcers perforation is sometime an optional modality. This case series purpose to identify such a case that response to conservative treatment. The success rate of conservative treatment in the present study was 94.7% suggested that inclusion criteria were important to distinguish minor perforation from major perforation. Same as the study of Gracias et al. [6], we found that air and fluid leakage in small volume shown by free air found under diaphragm, from abdominal X-ray film, not more than the 1^st^ lumbar vertebral column width and no free fluid seen by bedside ultrasound (fluid < 250 ml) indicated successful non-operational treatment.

Another criterion was developed in our case series was the detection of how long peritoneal contamination was occurred. If large fluid volume loss was found on arrival reflected long time peritoneal contamination. Thus the total fluid resuscitation in the first day would be exceeding than 5 ml/kg/h. The present series displayed a total fluid used in the first 24 h not more than 5 ml/kg/h was another good criterion. In addition, strictly placed nasogastric tube to decompress stomach and duodenum was also significant factor.

Conservative management was also designed for colonic diverticulitis patient with significant pain or localized peritonitis but absence of perforation. Determine outcomes of management on the 2nd day after admission to prevent delay operation. Complication and clinical course support that patients include in criteria had less complications and better clinical outcomes. Conservative group in our case series had shorter average length of hospital stay when compare to the study of Matsuda et al. [7] on laparoscopic omental patch to repair perforation of peptic ulcer (17 days) and the study of Antonio et al. [8] (9 ± 4 days) and compare with open omental patch [7] (17.3 days). Conservative treatment had no wound complication but questionable on band adhesion. However, conservative treatment had complication of prolong fever and pleural effusions. The opposition of conservative treatment was doubtful diagnosis. In this study, 20 cases were clinically suspect peptic ulcer (history of peptic ulcer symptoms in 12 cases and history of NSAID used in 8 cases) without prove. This study did not design other diagnostic imaging for selected patient, so it could not make a definitely diagnosis of perforated peptic ulcer. The author suggested that further in-depth prospective study using criteria for conservative treatment patient with perforated peptic ulcer is necessary and useful.

## 5 Conclusions

Conservative non-surgical management in pneumoperitoneum-peritonitis patients who were suspected of peptic ulcers perforation by using good criteria to select patients could be successful with shorter hospital stay and decrease the number of patient that need an operation.

## Conflicts of interest

No.

## Sources of funding

Surgical Department, Songkhla Hospital, Songkhla, Thailand.

## Ethical approval

this study was permit from ethical approval from Hospital Director of Songkhla hospital. included permission to publish of my study

## Consent

Every case has individual inform written and signed consent for treatment and publication in academic study.

## Author’s contribution

No other contributors.

## Registration of research studies

Registration at Thai Clinical Trials Registry (TCTR) www.thaiclinicaltrials.in.th/ <thaiclinicaltrials@gmail.com>.

My study TCTR identification number is TCTR20190111005.

## Guarantor

DR. Pongsatorn Asanasak.

## Provenance and peer review

Not commissioned, externally peer-reviewed.
